# Simultaneous Coproduction of Hydrogen and Ethanol in Anaerobic Packed-Bed Reactors

**DOI:** 10.1155/2014/921291

**Published:** 2014-09-11

**Authors:** Cristiane Marques dos Reis, Edson Luiz Silva

**Affiliations:** Department of Chemical Engineering, Federal University of São Carlos, Washington Luis Road, km 235, 13565-905 São Carlos, SP, Brazil

## Abstract

This study evaluated the use of an anaerobic packed-bed reactor for hydrogen production at different hydraulic retention times (HRT) (1–8 h). Two reactors filled with expanded clay and fed with glucose (3136–3875 mg L^−1^) were operated at different total upflow velocities: 0.30 cm s^−1^ (R030) and 0.60 cm s^−1^ (R060). The effluent pH of the reactors was maintained between 4 and 5 by adding NaHCO_3_ and HCl solutions. It was observed a maximum hydrogen production rate of 0.92 L H_2_ h^−1^ L^−1^ in R030 at HRT of 1 h. Furthermore, the highest hydrogen yield of 2.39 mol H_2_ mol^−1^ glucose was obtained in R060. No clear trend was observed by doubling the upflow velocities at this experiment. High ethanol production was also observed, indicating that the ethanol-pathway prevailed throughout the experiment.

## 1. Introduction

Hydrogen produced during acidogenesis stage of the anaerobic digestion is of significant interest, commercially and environmentally. It is also considered a clean fuel and, thus, it does not release greenhouse gases during combustion. However, to produce hydrogen from organic waste anaerobically, it is important to eliminate the methanogenic stage of the process by inactivating microorganisms responsible for methane conversion. It is necessary to adopt different operating strategies because the reactional environment should adapt in response to the condition applied. Some methods of inhibiting methanogenesis include pretreating the inocula to inactivate the methanogens and maintaining the pH of the system at specific values.

Furthermore, the choice of the reactor to produce hydrogen anaerobically is an important factor in optimizing hydrogen production. Good mixing conditions and high microbial biomass retention are desirable, and thus the right reactor configuration is essential. Attached growth reactors such as anaerobic packed-bed reactors (APBRs) appear to provide the best conditions. These reactors have a larger surface area available for microorganism deposition, and as a result, they are often used in research on hydrogen production [1–9].

Regarding APBR, various aspects have already been investigated. Some of these aspects include substrate, pH, temperature, inoculum, and support material for biomass adhesion. Several studies have investigated APBR with various sources of carbon, including sucrose [1, 2, 8], glucose [3, 5, 6, 9–11], domestic or industrial wastewater [12, 13], synthetic industrial paper effluent [14], palm extraction oil [15], and mixed fruit peel [16].

Studies in batch reactors [17, 18] have shown that pH is crucial for hydrogen production and metabolite formation. However, there is no consensus on the ideal pH for hydrogen production. In APBR, some researchers have opted to work in the 6-7 pH range [2, 4, 9] whereas others have preferred not to change the feed solution leading to a pH between 5 and 6 [1, 3, 5, 8]. Another important aspect in studies on APBR is related to the selection of the support material for biomass adhesion. Activated carbon [1, 2], packing rings [8], polyurethane foam [9], expanded clay [1, 5] are some of the materials that have been employed in recent studies.

Because of the diversity of parameters adopted by researchers, it appears to have no agreement about the best operation ranges to produce hydrogen. Even in studies that employ the same carbon source (glucose) and support material (expanded clay) as we did, the reported results have differed [1, 5].

Studies in attached-growth reactors, specially in fluidized-bed reactors [19–21], suggested that hydrodynamic factors may have significant results in hydrogen production since good mixing conditions can favor mass transfer among phases in anaerobic digestion. Parameters such as upflow velocity, effluent recycle rate, and porosity are among the factors that could be studied in order to reach higher hydrogen production rates. However, there is still a lack about the impact of hydrodynamics impacts in packed-bed reactors.

As a category of attached-growth reactors, APBR shows as primary feature a good biomass retention. The use of immobilized inoculum helps to create a stable environment for hydrogen production. Enhancing the mass transfer between biofilm and bulk, for instance, appears to have an important role in anaerobic digestion. The adjustment of the hydrodynamic parameters turns in a key point in hydrogen production in attached-growth reactors. Therefore, to contribute to a better operational understanding of anaerobic packed-bed reactors in hydrogen production, a study was conducted to investigate the influence of HRT and upflow velocity on hydrogen production in two reactors filled with expanded clay and fed with synthetic wastewater containing glucose as carbon source (approximately 3500 mg L^−1^).

## 2. Materials and Methods

### 2.1. Anaerobic Packed-Bed Reactors

**Figure 1:**
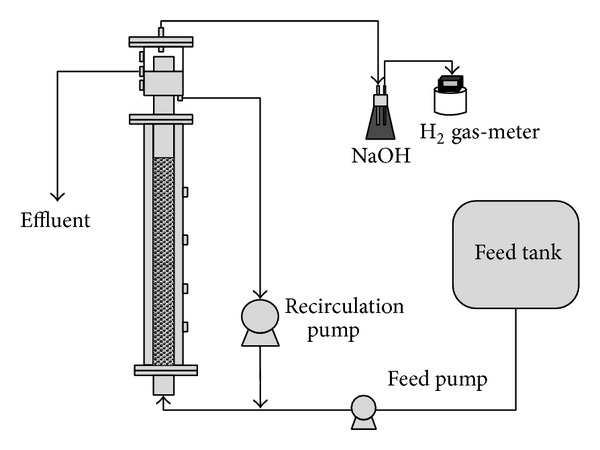
Schematic description of anaerobic packed-bed reactor (APBR).

The study employed two APBRs constructed from acrylic (5.3-cm diameter and 190-cm height, each) and filled with expanded clay (diameter = 2.8–3.3 mm and density = 1.5 g cm^−3^).  Figure 1 shows a basic outline of the process employed.

The inoculum was adapted to the reactors under batch mode for 48 hours, and following the 48-hour period, the reactors operated under continuous mode. The reactors began operation at HRT of 8 h, which was subsequently reduced to 1 h. The HRT was reduced when hydrogen production and glucose conversion stabilized. The choice of upflow velocities (*V*_up_) was selected based on the minimum fluidization velocity (*V*_mf_, for expanded clay: *V*_mf_ = 1.24 cm s^−1^). The reactors were named based on the velocity at which they were operated. R030 is the reactor with *V*_up_ of 0.30 cm s^−1^ (24% of *V*_mf_), and R060 is the reactor with *V*_up_ of 0.60 cm s^−1^ (48% of *V*_mf_). R030 and R60 operated continuously for 217 days.

### 2.2. Synthetic Wastewater and Inoculum

The APBRs were fed with synthetic wastewater that contained glucose at a concentration of 3500 mg L^−1^. The nutrient concentrations were as follows (in mg L^−1^): CO(NH_2_)_2_ (125); NiSO_4_ *·*6 H_2_O (1); FeSO_4_ *·*7 H_2_O (5); FeCl_3_ *·*6H_2_O (0.5); CaCl_2_ *·*6H_2_O (47.0); CoCl_2_ *·*2H_2_O (0.08); SeO (0.07); KH_2_PO_4_ (85.0); K_2_HPO_4_ (21.7); and Na_2_HPO_4_ *·*2H_2_O (33.4) [22]. Hydrochloric acid (30%) and sodium bicarbonate (0.84 g L^−1^) were also added as buffer solutions to maintain the pH in the reactors at 4-5. Reactors inoculation was performed only once during the first 48 h with sludge from a treatment plant for swine waste. The H_2_ productivity of the sludge was enhanced by heat treatment [23]. The reactors were inoculated at a rate of 3.5% of sludge feed volume.

### 2.3. Chemical Analyses

The chemical oxygen demand (COD), pH, and solids (total solids, TS; volatile suspended solids, VSS; and total volatile solids, TVS) were measured in accordance with standard methods [24]. The glucose concentration was measured with an enzymatic GOD-PAP method [25].

The biogas hydrogen content was determined by gas chromatography (GC-2010, Shimadzu, Japan) using a thermal conductivity detector (TCD) with argon as the carrier gas, and the column was packed with Supelco Carboxen 1010 Plot (30 m × 0.53 mm i.d.) [26]. A gas meter (Type TG1; Ritter Inc., Germany) was used to measure the amount of hydrogen generated.

The concentrations of volatile fatty acids (VFA) and alcohols were also measured using a gas chromatography system (GC-2010, Shimadzu, Japan) that was equipped with FID and COMBI-PAL headspace injection (AOC 5000 model) and a HP-INNOWAX column (30 m × 0.25 mm i.d. × 0.25 *µ*m film thickness) [26].

## 3. Results and Discussion

### 3.1. Effect of HRT and Upflow Velocity on H_2_ Production

**Figure 2:**
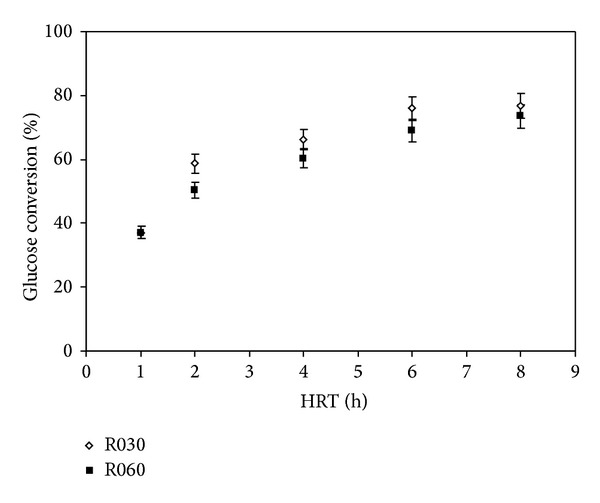
Effect of HRT on glucose conversion in R030 and R060.

Figure 2 shows glucose conversion as a function of the HRT variation in the reactors. Glucose conversion was calculated as [influent glucose concentration, effluent glucose concentration] per influent glucose concentration. Each reactor operated at a different upflow rate to facilitate the analysis of the operating behavior at different HRTs.

As shown in Figure 2, throughout operation, R030, which was under an upflow velocity of 0.30 cm s^−1^, presented similar conversion rates compared to R060, which operated at 0.60 cm s^−1^, when taking in account the deviations. HRT of 2 h was an exception; at this HRT, R030 was slightly more efficient than R060. The maximum conversion rates were achieved in both reactors at HRT 8 h, and the conversion rates dropped when the HRT decreased. Conversion rates ranged from 30.4% to 80.0% in R030 and from 33.1% to 77.9% in R060. The conversion rate decreased greatly at HRT 1 h. It is possible that the substrate residence time in the reactor was shorter than that required for organic matter degradation, leading to a reduction in glucose conversion in expanded bed reactors at HRT 1 h just like it was observed by De Amorim et al. [27] in fluidized-bed reactor when glucose concentration was elevated. The results obtained in our study are in agreement with literature data for other studies in APBR regarding HRT 1 h.

**Figure 3:**
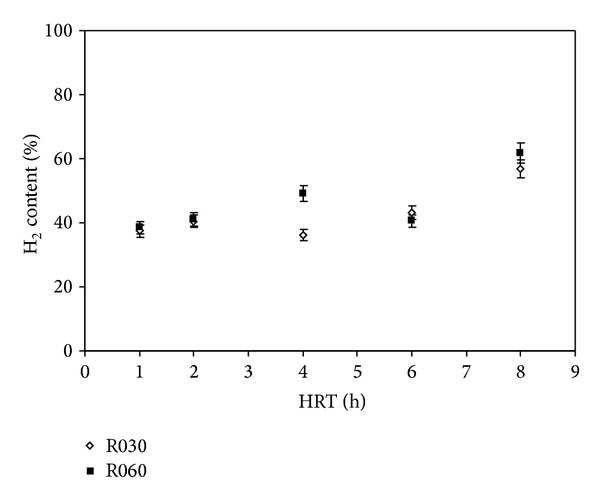
Effect of HRT on H_2_ content in R030 and R060.

Figure 3 shows H_2_ content in R030 and R060. Biogas was composed of H_2_ and CO_2_. Methane was not detected. For all HRT applied, with the exception of HRT 6 h, R060 showed slightly higher H_2_ concentrations in the biogas than R030. The H_2_ concentration was maximal at HRT of 8 h for both R030 (56.8%) and R060 (61.8%). When HRT was 8 h or higher, the H_2_ concentration was reduced, appearing to stabilize at subsequent HRT. The minimum values achieved were 37.4% and 38.5% for R030 and R060, respectively.

The H_2_ and CO_2_ produced are released from the water medium into the gas phase. At first, the beneficial effect of the upflow velocity on mass transfer parameters is not conclusive. The presence of H_2_ in biogas generated in our work agrees with studies available in the literature on APBR. Chang et al. [1] obtained H_2_ content in biogas ranging from 9.5% to 45.8%. Lee et al. [2] showed that this content varied between 30% and 40%, while Li et al. [8] reported the H_2_ content in biogas to be between 28.5% and 40.8%. Other studies have achieved higher concentrations. Zhang et al. [3] obtained 74% H_2_ content, and Leite et al. [5] reported values ranging from 75% to 90%.

**Figure 4:**
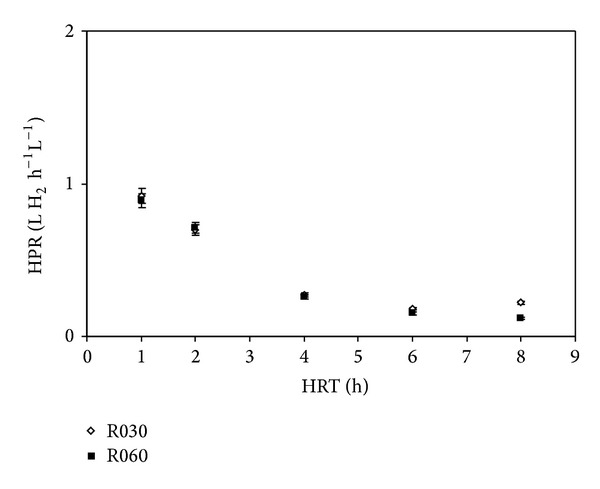
Effect of HRT on hydrogen production rate in R030 and R060.

Figure 4 shows the hydrogen production rate (HPR) as a function of HRT in R030 and R060. HPR was calculated as liters of hydrogen produced per hour per reactional volume of the reactor.

As shown in Figure 4, both reactors show an increase in HPR due to a decrease in HRT from 8 h to 1 h. HPR remained stable with only a slight variation between HRT 8 h and 4 h. However, from HRT of 4 h to 1 h, HPR increased in both reactors.

According to Chang et al. [1] and Lee et al. [2], the presence of suspended cells between bed particles (voids) favors hydrogen production because it allows for microbial growth. However, the nature of the particle bed allows for good retention of biomass in the form of biomass or as extracellular polymeric substances. Still, empty space in the reactor may be increased by increasing the upflow rate applied to the particle bed [1, 2]. Furthermore, Kumar and Das [28] investigated a pure culture of IIT-BT 08* Enterobacter cloacae* for hydrogen production by varying the recycling rate and observed that an increase in the recycle rate led to an increase in hydrogen production due to the reduction of the resistance to mass transfer. Also studying the recycle rate on hydrogen production, Ngoma et al. [19] verified in fluidized-bed reactors with an external gas-disengager that an increase in the recycle rate (1.3 to 3.5 L min^−1^) leads to an increase in the H_2_ productivity (2.1 to 8.7 L H_2_ h^−1^ L^−1^) under 45°C. Same effect was obtained under 70°C (2.8 to 14.9 L H_2_ h^−1^ L^−1^). According to the authors, vigorous mixing process within the gas-disengager due to high rates of effluent recycling permits an efficient removal of undissolved or nonsolibilized H_2_ that may be present in the effluent. This gas removal associated with the enhanced mass transfer induces the hydrogen production. Also in fluidized-bed reactors, Dos Reis and Silva [20] verified that it should have an optimum upflow velocity range that could maximize the hydrogen production. According to them, due to the good mixing conditions inside the reactors resulted from a high velocity (1.24 cm s^−1^) they obtained a hydrogen production rate of 2.21 L H_2_ h^−1^ L^−1^. In another research, Obazu et al. [21], when testing the relation between the reactor volume and the recycle rate in fluidized-bed reactors, also verified that the high degree of fluid turbulence is good for hydrogen release, enhancing hydrogen production.

These findings should be considered when employing different upflow velocities in face of the increase of the turbulent conditions. Thus, because R060 was operated at a rate twice as high as that of R030, it was capable of presenting better hydrogen production results. However, quantitative results show that the HPR ranged from 0.22 to 0.92 L H_2_ h^−1^ L^−1^ in R030 but only from 0.12 to 0.89 L H_2_ h^−1^ L^−1^ in R060.

The divergence between the expected results and our results indicates that the upflow rate range employed in our study had no effect on hydrogen production. Maybe a higher upflow velocity till the limit of the minimum fluidization velocity would be more suitable for analyzing this parameter. R030 and R060 presented lower HPR than those reported by Chang et al. [1] (1.32 L H_2_ h^−1^ L^−1^ at HRT 2 h), Lee et al. [2] (7.4 L H_2_ h^−1^ L^−1^), and Jo et al. [9] (0.3 L H_2_ h^−1^ L^−1^) but higher than those reported by Li et al. [8] (0.26 L H_2_ h^−1^ L^−1^ at HRT 2 h). The literature indicates that a small applied HRT results in a high HPR.

**Figure 5:**
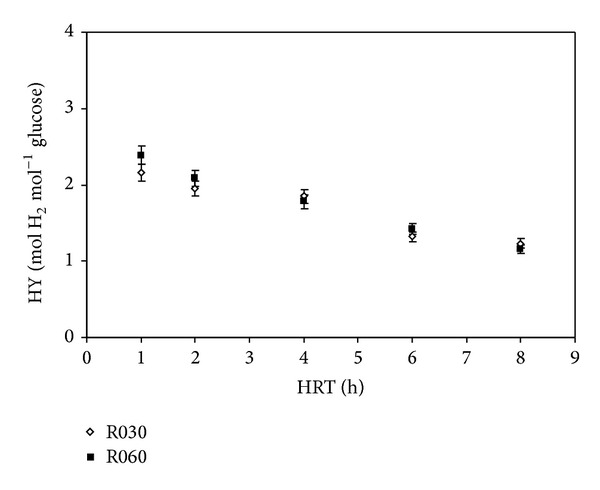
Effect of HRT on hydrogen yield in R030 and R060.

Figure 5 shows the hydrogen yield (HY) as a function of HRT in R030 and R060. HY was calculated as moles of hydrogen produced per mole of glucose converted.

Figure 5 shows that hydrogen yield values and the behavior of HPR for the two reactors were similar. The yield values for the reactors were similar when HRT decreased. This result indicates that the rates adopted for the APBR in question did not enable us to clearly identify a positive influence of increasing upflow velocities on hydrogen production. In general, HY increased when HRT decreased, with the highest yield occurring at HRT 1 h for both reactors.

When examining the combined results of HPR and HY, it appears that at HRT 1 h, R060 performed best with regard to H_2_ production. When comparing our results to other studies, the HY values obtained in our work with R030 (1.23 mol H_2_ mol^−1^ glucose to 2.16 mol H_2_ mol^−1^ glucose) and R060 (1.16 mol H_2_ mol^−1^ glucose to 2.39 mol H_2_ mol^−1^ glucose) are in agreement with HY values obtained in APBR. Chang et al. [1] obtained yield values ranging from 0.08 mol H_2_ mol^−1^ sucrose to 1.14 mol H_2_ mol^−1^ sucrose; the peak was observed at HRT 2 h. However, Lee et al. [2] reported HY values between 2.9 mol H_2_ mol^−1^ sucrose and 3.9 mol H_2_ mol^−1^ sucrose when HRT varied from 0.5 h to 4 h. Li et al. [8] obtained HY values from 0.78 mol H_2_ mol^−1^ glucose to 1.22 mol H_2_ mol^−1^ glucose with maximum and minimum HY at HRT of 14 h and 2 h, respectively.

The hydrogen production results vary greatly due to different experimental conditions such as microorganism cultures, substrates, and pH values.  Table 1 summarizes the results from several studies on APBR to show this diversity.

The acetic pathway illustrated by the following indicates that four moles of hydrogen are produced for each mol of glucose degraded:

(1) $$\begin{array}{c} {\text{C}}_{6}{\text{H}}_{12}{\text{O}}_{6}+2{\text{H}}_{2}\text{O}⟶2{\text{CH}}_{3}\text{COOH}+2{\text{CO}}_{2}+4{\text{H}}_{2} \end{array}$$

The acetic pathway is deemed the most effective pathway for hydrogen production. If this pathway is used as a reference, the highest HY value obtained so far was reported by Lee et al. [2] at HRT of 0.5 h. They reported 49% of the highest theoretical HY for sucrose (8 mol H_2_ mol^−1^ sucrose). However, this yield is lower than what was obtained by Leite et al. [5] who reported 62% (2.39 mol H_2_ mol^−1^ glucose) of the maximum theoretical value for glucose (4 mol H_2_ mol^−1^ glucose).

Table 1 also provides the range of upflow velocities employed by the studies. The best yield was presented by Lee et al. [2] when the upflow rates adopted ranged between 0.001 cm s^−1^ and 0.01 cm s^−1^, with the best results at 0.01 cm s^−1^. However, Li et al. [8] who also worked with sucrose and a mixed culture at upflow velocities between 0.03 cm s^−1^ and 0.39 cm s^−1^ showed a lower yield than that obtained by Lee et al. [2].

However, our study with glucose as the substrate and employing higher upflow rates than Lee et al. [2] obtained higher H_2_ yields. Leite et al. [5], who also employed glucose as the substrate and used expanded clay as the support material for biomass adhesion, showed H_2_ yields similar to ours, even though we adopted a higher range of upflow rates.

The effect of upflow velocity applied to reactors for APBR is not relevant when it comes to increasing or decreasing H_2_ production. Other aspects, such as adopted substrate and support material and/or chosen type of inoculum pretreatment, appear to have a higher influence on H_2_ production. Furthermore, based on the literature reported in Table 1, the highest hydrogen production values are achieved at the shortest HRT. However, yield values do not show this trend [8], although H_2_ yield increased as applied HRT decreased in our study.

### 3.2. Influence of HRT and Upflow Velocity on Production of VFA and Alcohols

Table 2 shows the distribution of soluble metabolite products (SMP) generated in R030 and R060 in relation to the HRT. The main SMPs were ethanol (EtOH) and acetic acid (HAc). Butyric acid (HBu), propionic acid (HPr), and methanol (MetOH) were also generated.

The production of metabolites did not vary significantly with regards to the upflow velocity range used, resulting in similar concentrations for R030 and R060. Thus, the use of different upflow velocities does not necessarily lead to different configurations in SMP distribution. Our results indicated that EtOH production was favored over HBu production, which is commonly associated with hydrogen production. HAc production was also relevant throughout our study.

The HAc/HBu ratio is commonly used as an indicator of bioprocess efficiency in hydrogen production. Although this parameter increased as HRT was decreased in both R030 and R060, it should not be used as the only parameter to indicate H_2_ production effectiveness. HAc production prevailed over HBu production, which decreased when HRT changed. The low presence of HBu compared to EtOH and HAc which indicates the presence of a metabolic pathway to produce hydrogen differs from that of HAc and HBu because there was no H_2_ production decrease when HBu production declined. HAc production remained constant when the HRT was changed. However, EtOH production increased drastically from 31% to 57% in R030 and from 29% to 50% in R060 when the HRT was decreased from 6 h to 4 h.

This change in metabolites occurred when there was a decrease in HBu production in the reactors, indicating that the HBu pathway was favored over the EtOH pathway. Because HAc production varied only slightly, it is suggested that H_2_ production occurred simultaneously with HAc production. Ren et al. [29] presented a way of producing H_2_ in which one mol of HAc, two moles of H_2_, and one mol of EtOH were generated for each mol of glucose degraded, as shown in the following:

(2) $$\begin{array}{c} {\text{C}}_{6}{\text{H}}_{12}{\text{O}}_{6}+{\text{H}}_{2}\text{O}⟶{\text{C}}_{2}{\text{H}}_{5}\text{OH}+{\text{CH}}_{3}\text{COOH}+2{\text{H}}_{2}+2{\text{CO}}_{2} \end{array}$$

Based on the traditional pathway of EtOH production from glucose, H_2_ production was expected to decrease due to increased production of solvents [29]. However, in both reactors in our study, the H_2_ production increased up to HRT 1 h. It is unclear what may have caused this change in metabolic pathway. Zhu et al. [30] worked with a batch reactor and glucose to study the metabolic pathways as a function of pH. They found that the presence of different metabolic pathways depended on the pH range adopted and that the production of organic acids increased at pH values between 5.5 and 6.0.

However, EtOH production did not depend on the pH range adopted. The production of metabolites was limited at pH values lower than 4.5. Furthermore, Zhu et al. [30] found EtOH and H_2_ production at pH 5.5. At this pH, the main metabolic pathways present in their study were mainly conducive to HAc, EtOH, and H_2_ production. As in our study with APBR, there was simultaneous production of Hac, EtOH, and H_2_. These results confirm that EtOH production without decreasing H_2_ production is possible as shown in (2) proposed by Ren et al. [29].

Our results also indicate that the HAc/EtOH ratio shows the variation in the metabolic pathway for the main products obtained. Furthermore, these data also point to the production of HAc and especially EtOH, which always prevailed over the HAc production (HAc/EtOH < 1). The ethanolic pathway predominated at HRTs ranging from 6 h to 4 h. When the HRT was reduced to 2 h, the HAc production became dominant again in both R030 and R060. The highest production of HAc and H_2_ occurred at HRT 1 h. Furthermore, this phase was also the only one in which HAc production prevailed over EtOH production.

Table 3 shows the data for production of hydrogen, organic acids, and alcohols in several studies with APBR. These data refer to metabolites produced at maximum H_2_ production. EtOH production did not prevail in any of these studies, with the exception of Wu et al. [4], who obtained 60% EtOH along with other SMPs.

The predominant metabolites generated were HAc and HBu. Chang et al. [1] obtained low HBu production when H_2_ production peaked, and EtOH appeared to be one of the major SMPs generated. This result, combined with the results obtained for R030 and R060, shows that butyric and ethanolic pathways compete with each other, while HAc production and EtOH production occur simultaneously or use the same metabolic pathway.

Table 3 shows the distribution of generated metabolites in diverse studies in packed-bed reactors compared to the present study.

The results of Chang et al. [1] indicate that an increase in upflow rate leads to a decrease in ethanol production. However, Lee et al. [2] suggest that an increase in upflow rate has no significant impact on the distribution of metabolites, which also appears to be true in the present study. Lee et al. [2] reported the highest production of volatile acids. However, at the HRT where production of volatile acids was the highest, the ethanol production was the lowest. Furthermore, the butyric pathway was favored when metabolite production was relevant.

## 4. Conclusions

The adoption of two different upflow rates 0.30 cm s^−1^ and 0.60 cm s^−1^ in APBR (R030 and R060, respectively) at varying HRTs was observed to have a nonsignificant influence on hydrogen production. Our results verified that a long HRT increased the volumetric hydrogen production obtained, with a maximum value of 0.92 L H_2_ h^−1^ L^−1^ obtained in R030. Hydrogen yield was highest in R060, reaching 2.39 mol H_2_ mol^−1^ glucose at HRT 1 h. The main metabolites generated were ethanol and acetic acid, indicating that the ethanol-type pathway prevailed throughout the experiment.

## Figures and Tables

**Table 1:** Comparison of studies in packed-bed reactors for hydrogen production.

Reference	Substrate/ concentration (g L^−1^)/ ORL (kgDQO*·*m^−3^ *·*d^−1^)	Inoculum/pretreatment method	Support material	*V*_up_ (cm s^−1^)	Temperature (°C)	pH	HRT (h)	HY (mol H_2_ *·*mol^−1^ substrate)	HPR (L L^−1^ h^−1^)
Chang et al. [1]	Sucrose 17.8/85–854	Municipal sewage sludge Acid	Expanded clay	—	35.0	6.7	0.5–5.0	0.1–1.3∗	0.2–0.4

Chang et al. [1]	Sucrose 17.8/213–854	Municipal sewage sludge Acid	Activated carbon	—	35.0	6.7	0.5–2.0	0.5–1.4∗	0.6–1.3

Lee et al. [2]	Sucrose 17.8/106–854	Municipal sewage sludge Acid	Activated carbon	—	35.0	6.7	0.5–4.0	2.9–4.0∗	1.2–7.4

Zhang et al. [3]^a^	Glucose 10.5/194	*Clostridium acetobutylicum *	Glass beads	0.005∗	30.0	4.9	1.3∗∗	0.9	0.2

Wu et al. [4]	Sucrose 17.8/107	Municipal sewage sludge Acid	Polyethylene-octane elastomer (POE)	—	35.0	6.0	4.0	0.4	0.3∗

Wu et al. [4]	Glucose 18.9/113	Municipal sewage sludge Acid	Polyethylene-octane elastomer (POE)	—	35.0	6.0	4.0	0.7	0.4∗

Wu et al. [4]	Fructose 18.9/113	Municipal sewage sludge Acid	Polyethylene-octane elastomer (POE)	—	35.0	6.0	4.0	0.6	0.2∗

Leite et al. [5]	Glucose 2/96	Mixed culture Natural fermentation	Expanded clay	0.024	30.0	3.9–7.3	0.5	1.8–2.5	—

This study	Glucose 3.5/10–84	Swine slaughterhouse sludge Heat-shock	Expanded clay	0.3	25.0	4-5	1.0–8.0	1.2–2.2	0.2–0.9

This study	Glucose 3.5/10–84	Swine slaughterhouse sludge Heat-shock	Expanded clay	0.6	25.0	4-5	1.0–8.0	1.2–2.4	0.1–0.9

∗Based on article data, ^a^unsaturated flow reactor, and ∗∗HRT = reactor volume/influent flow rate.

**Table 2:** Distribution of generated metabolites as a function of HRT for R030 and R060.

APBR	HRT (h)	EtOH/SMP (%)	HAc/SMP (%)	HPr/SMP (%)	HBu/SMP (%)	MetOH/SMP (%)	TVFA (mmol L^−1^)	SMP (mmol L^−1^)	HAc/HBu∗	HAc/EtOH∗
R030	8	32.7	31.8	0.3	28.8	6.5	18.8	30.9	1.1	0.9
	6	31.7	29.1	15.0	15.6	8.6	19.3	32.3	1.9	0.9
	4	57.5	21.1	9.7	4.6	7.1	17.3	48.7	4.7	0.4
	2	59.7	18.5	9.7	3.0	9.1	14.6	46.9	6.2	0.3
	1	39.2	36.5	11.5	3.9	8.8	19.8	38.1	9.3	0.9

R060	8	33.0	29.6	2.9	27.5	6.9	14.9	24.9	1.2	0.9
	6	29.1	25.2	23.5	14.3	7.9	18.9	29.9	1.8	0.9
	4	50.5	25.8	12.3	5.2	6.3	20.4	47.1	4.9	0.5
	2	40.1	27.0	21.7	5.0	6.2	11.0	20.5	5.4	0.7
	1	36.5	39.4	13.1	5.2	5.9	20.2	35.1	7.6	1.2

TVFA: total volatile fatty acids, TVFA = HAc + HBu + HPr, and SMP = TVFA + EtOH.

∗Molar ratio.

**Table 3:** SMP distribution in studies using APBRs for hydrogen production.

Reference	Substrate (support material)	HRT (h)	EtOH/SMP (%)	HAc/SMP (%)	HPr/SMP (%)	HBu/SMP (%)	MetOH/SMP (%)	HVl/SMP (%)	HCpr/SMP (%)	TVFA (mmol L^−1^)	SMP (mmol L^−1^)	HAc/HBu∗	HAc/EtOH∗
Chang et al. [1]∗∗	Sucrose (expanded clay)	5	46.3	27.2	17.4	9.1	—	—	—	29.9	55.8	2.9	0.6
		2	42.7	24.9	24.6	7.8	—	—	—	44.5	77.7	3.2	0.6
		1	25.3	31.1	33.9	9.7	—	—	—	50.7	67.9	3.2	1.2
		0.5	19.5	29.4	39.3	11.7	—	—	—	64.2	79.9	2.5	1.5

Lee et al. [2]∗∗	Sucrose (activated carbon)	4	1.6	31.3	36.8	29.7	—	0.6	—	94.9	96.5	1.1	19.3
		2	21.0	20.8	16.6	37.8	—	3.9	—	89.2	112.8	0.6	1.0
		1	9.6	19.2	23.4	41.8	—	6.0	—	147.4	163.0	0.5	2.0
		0.5	10.3	20.6	24.7	41.2	—	3.2	—	136.2	151.8	0.5	2.0

Zhang et al. [3]	Glucose (glass beads)	1.3∗∗∗	—	70.6	—	29.4	—	—	—	1.7	1.7	2.4	—

Wu et al. [4]	Fructose (POE)	4	52.8	25.1	10.1	12.1	—	—	—	355.7	616.3	2.1	0.5

Wu et al. [4]	Sucrose (POE)	4	62.0	29.9	1.9	6.2	—	—	—	605.7	1302.2	4.8	0.5

Wu et al. [4]	Glucose (POE)	4	69.2	22.3	1.3	7.1	—	—	—	418.5	1064.4	3.1	0.3

Leite et al. [5]	Glucose (expanded clay)	0.5	—	57.9	3.0	34.0	—	—	5.1	12.9	12.9	1.7	—

HVl: valeric acid. HCpr: caproic acid.

∗Molar ratio, ∗∗based on article data, and ∗∗∗HRT = reactor volume/influent flow rate.
